# Perceptions of eHealth and digitalization among professional anaesthesia personnel: A Swedish national study

**DOI:** 10.1111/aas.14587

**Published:** 2025-01-30

**Authors:** Pether Jildenstål, Camilla Viseu, Kristian Hermander, Carina Sjöberg, Katarina Hallén, Randolph Schnorbus, Annelie Augustinsson

**Affiliations:** ^1^ Care in High Technological Environments, Department of Health Sciences Lund University Lund Sweden; ^2^ Department of Anaesthesiology and Intensive Care Skåne University Hospital Lund Sweden; ^3^ Institute of Health and Care Sciences, Sahlgrenska Academy University of Gothenburg Gothenburg Sweden; ^4^ Department of Anaesthesia and Intensive Care, Institute for Clinical Sciences, Sahlgrenska Academy University of Gothenburg Gothenburg Sweden; ^5^ Department of Anaesthesiology, Surgery and Intensive Care Sahlgrenska University Hospital Gothenburg Sweden; ^6^ Department of Anaesthesiology and Intensive Care, Örebro University Hospital and School of Medical Sciences Örebro University Örebro Sweden

**Keywords:** anaesthesia, digitalization, eHealth, surgery

## Abstract

**Background:**

The objective of this study was to evaluate anaesthesia care professionals' perceptions and attitudes regarding the implementation and advancement of digital solutions in perioperative care.

**Methods:**

Anaesthesia personnel working in public Swedish institutions where anaesthesia is administered were invited to respond to an online survey regarding their attitudes towards digitalization in the workplace and their perceptions of information provision and future digitalization within anaesthesia and surgical healthcare. Data were analyzed using descriptive statistics, independent‐samples Kruskal–Wallis tests, and post‐hoc pairwise comparisons.

**Results:**

The survey response rate was 64.0% (*n* = 627). Most respondents agreed/strongly agreed that digital solutions facilitate their work, the preoperative preparation, patient participation, and being involved in the patients' journeys throughout the perioperative care process. The majority also agreed/strongly agreed that digital solutions could make more patients adequately prepared before anaesthesia/surgery, reduce the number of non‐optimized patients, and adapt the perioperative process to the patients' individual needs, as well as lead to reduced costs for the healthcare provider and reduced cancelled anaesthesia/surgeries. However, there were statistically significant differences between responses in relation to age groups, where the largest differences were observed between respondents in the age groups 20–30 and 61–70 years and in relation to what part of Sweden respondents worked in, with the largest differences between respondents working in Southern Sweden and the middle part of Sweden.

**Conclusion:**

Swedish anaesthesia personnel are confident that digital solutions may enhance the efficiency of care within the anaesthesia setting. However, varying perceptions on the benefits and necessity of digital solutions are indicated.

**Editorial Comment**

These findings show that there are positive attitudes towards digital solutions amongst Swedish anaesthesia care professionals regarding the benefits for their own workflow. The findings also show positive expectations on the patients' behalf regarding improvement in patient care from digital solutions. Further study might focus on documenting how and to what extent use of digital solutions actually benefits patients.

## 1 INTRODUCTION

In recent decades, the duration of inpatient hospital care for planned major surgery has decreased extensively due to the introduction of fast‐track programs and use of various perioperative interventions.^1^ Due to time restraints, patients are increasingly required to manage their pre‐ and postoperative self‐care on their own to a larger extent.^2^ As patients are expected not only to participate in their pre‐ and postoperative care, but also to take greater responsibility for their self‐care,^3^ the development of digital health services within this context has also accelerated.

The designation digital health comprises a systematic use of information and communications technologies, computer science, and data to support both patients' and health workers' decision‐making to strengthen disease resilience and improve health. Digital solutions can be used to exchange important health information and monitor symptoms, as well as increase interaction, communication, and collaboration between patients and healthcare professionals.^4^, ^5^, ^6^ The benefits of digitalization include the ability to create a healthcare system characterized by improved effectiveness, enhanced safety, and reduced costs, as well as to empower patients, which may lead to increased self‐care participation.^7^ The benefits associated with integrating digital technology in the healthcare sector to foster environmental sustainability and mitigate carbon dioxide emissions have also been a subject of discourse.^8^ However, the expansion of digital tools has increased cybersecurity risks, leaving organizations more vulnerable to cyberattacks, data breaches, and ransomware. Privacy concerns have also been intensified, as extensive data collection frequently occurs without full user awareness or consent. In healthcare, digitalization can exacerbate disparities among personnel directly involved in patient care, affecting their ability to use digital tools effectively and their influence on recipients, such as patients. This impacts patient safety, as difficulties in managing systems (e.g., apps) can lead to challenges in care delivery.^9^ Additionally, digitalization can reinforce social inequalities and create a digital divide, particularly in older individuals with less digital literacy^10^ and in rural areas with limited internet access.^9^

With the prevalence of digitalization in present times, research has been conducted on the topic within public administration, for example, e‐government and new digital healthcare systems.^11^, ^12^ The development of various digital eHealth services, that is, healthcare services supported by digital processes or technology,^13^ is constantly evolving. For instance, web‐based platforms, mobile phone applications, and use of text messages have significantly accelerated to facilitate access to information, communication, data collection, patient monitoring, and health and lifestyle management.^14^, ^15^, ^16^ The term eHealth not only characterizes a technical development, but also a new way of working, an attitude and a commitment for networked global thinking to improve healthcare by using information and communication technology.^4^ Hence, the workplace environment significantly impacts the successful implementation of new technical elements and staff support plays a crucial role in this process.^17^, ^18^, ^19^

To effectively integrate digital advancements in the healthcare arena, it is essential to consider the development and incorporation of these technologies.^20^ However, there is limited research conducted on how digitalization can enhance or change medical implications in line with scientific standards.^21^ The main objective of this study was to evaluate anaesthesia care professionals' perceptions and attitudes regarding the implementation and advancement of digital solutions in perioperative care.

## 2 METHODS

### 2.1 Data collection

A self‐report survey was developed to assess the research objective. During the study design process, no established instrument met the study's objectives or context. Therefore, a custom survey, inspired by the Knowledge, Attitude, and Practice (KAP) model^22^ was designed and pilot‐tested with seven nurses and five anaesthesiologists on two occasions to refine its structure and language.

After approval from the Swedish Ethical Review Authority (protocol number 2022‐06099‐01), contact information to professional anaesthesia personnel in Sweden, through websites or email addresses, were extracted from the Swedish Association for Anaesthesia and Intensive Care (SFAI) and the Swedish Association of Health Professionals registries of members, comprising nurse anaesthetists. A web‐based survey was subsequently distributed to 980 recipients on February 1, 2023. The survey remained accessible for a duration of 6 weeks, until March 17, 2023. During this period, reminders were sent to recipients who had not answered the survey on two occasions at 2‐week intervals. No incentive was provided for participation.

Before taking the survey, respondents were provided with an introduction regarding the content and aim of the study. This introduction explained that digitization in a technical context originally refers to the process of converting analogue data into a digital representation of information, such as by sampling sounds and other analogue electrical signals or by scanning images. It also described that digitalization involves integrating digital technologies into operations to optimize processes and enhance experiences. Furthermore, digital aids/solutions were defined as tools and technologies that utilize digital platforms or systems to support and improve various activities and processes, such as surgical planning programs and digital health declarations. Additionally, it was mentioned that more tools are under development, including functions such as reminding patients to stop taking certain medications and to shower twice before anaesthesia/surgery.

The survey comprised a total of 31 questions divided into four sections with inquiries related to demographics (*n* = 7), attitudes towards digitalization in the workplace (*n* = 5), perceptions of information provision in the workplace (*n* = 12), and perceptions of future digitalization within anaesthesia and surgical healthcare (*n* = 7). The questions regarding demographics included age at inclusion and work‐related inquires. The subsequent 24 questions were accompanied by 5‐point Likert scales, with 1 representing “strongly disagree,” 2 “disagree,” 3 “neutral,” 4 “agree,” and 5 “strongly agree.” An additional sixth option, “do not know,” was also included. There was no provision for free‐text responses.

### 2.2 Statistical analysis

Respondents' characteristics included age group (20–30, 31–40, 41–50, 51–60, 61–70 years), part of Sweden, that is, what part of Sweden the respondent worked in (south [south of Linköping], middle [between Linköping and Uppsala], north [north of Uppsala]), workplace (university hospital, regional hospital, private hospital), main practice (preoperative, daycare surgery, inpatient surgery, daycare and inpatient surgery, postoperative), work unit (<15 operating rooms, ≥15 operating rooms, private), work experience (<3, 3–6, 7–11, 12–14, >14 years), and first digital implementation in the workplace (<3, 3–6, 7–11, 12–14, >14 years).

All analyses were conducted using the IBM SPSS statistical computing package (version 28.0; SPSS, Inc., Chicago, Illinois, USA). Independent‐samples Kruskal–Wallis tests and post‐hoc pairwise comparisons with significance levels adjusted by the Bonferroni correction for multiple tests were conducted to evaluate the null hypothesis that there were no significant differences in study participants' responses to the scaled‐response questions based on age group affiliation and part of Sweden in which they worked. Statistics are shown as mean ± standard deviation (SD), sample average rank, and test statistic (adjusted for ties). Statistical significance was considered with a two‐tailed *p*‐value <.05. Pearsons Chi‐square test was conducted to evaluate the null hypothesis that there was no difference between age distribution in the subset of part of Sweden. Since the age distributions did not differ significantly from each other at the 0.05 level (*p* = .978), no multivariate comparisons were conducted.

## 3 RESULTS

Of a total of 980 recipients, 627 (64.0%) answered the survey. Approximately 83% of the respondents were between 31 and 60 years old. Almost half of the respondents worked in Southern Sweden, and the majority worked in regional hospitals with both daycare and inpatient surgery (Table 1). However, there were statistically significant differences between respondents (*p* <.001). In Southern Sweden, 37.8% worked in university hospitals, while the corresponding percentage for the middle of Sweden was 52.9% and for Northern Sweden 8.6%. In Northern Sweden, 91.4% worked in regional hospitals and none (0.0%) in private clinics. In the middle of Sweden, 11.9% worked in private clinics, and in Southern Sweden, the corresponding percentage was 3.3%.

**TABLE 1:** Basic characteristics of the survey respondents (*n =* 627).

Demographic or workplace characteristic	*n*	%
Age group, years		
20–30	28	4.5
31–40	171	27.3
41–50	168	26.8
51–60	180	28.7
61–70	80	12.8
Part of Sweden		
South	307	49.0
Middle	227	36.2
North	93	14.8
Workplace		
University hospital	224	38.9
Regional hospital	346	55.2
Private hospital	37	5.9
Main practice		
Preoperative	16	2.6
Daycare surgery	49	7.8
Inpatient surgery	92	14.7
Daycare and inpatient surgery	457	72.9
Postoperative	13	2.1
Work unit		
<15 Operating rooms	457	72.9
≥15 Operating rooms	146	23.3
Private unit	24	3.8
Work experience, years		
<3	62	9.9
3–6	125	19.9
7–11	107	17.1
12–14	73	11.6
>14	260	41.5
First digital implementation in the workplace, years		
<3	77	12.3
3–6	161	25.7
7–11	181	28.9
12–14	83	13.2
>14	125	19.9

Respondents' answers to the scaled‐response questions with answers ranging from strongly disagree (1) to strongly agree (5) and the sixth option of “do not know” are displayed in Table 2. Most respondents agreed/strongly agreed that digital solutions facilitate their work, as well as the preoperative preparation and patient participation. Additionally, most respondents felt that they are involved in the patient's journey throughout the perioperative care process. However, 238 (38.0%) did not know whether patients were well‐informed before the preoperative meeting with the anaesthetist. Most respondents disagreed/strongly disagreed to receiving information regarding the patient's recovery, rehabilitation, or possible side effects from the anaesthesia and/or surgery. More than one‐fourth did not know if patients easily can get in touch with the care provider if they have questions about the anaesthesia/surgery or the preoperative preparations, and more than half did not know whether patients receive good support after anaesthesia/surgery (e.g., exercise and diet). Furthermore, the respondents agreed/strongly agreed that it could be beneficiary if the patients are able contact the healthcare provider in a simple way both before and after the anaesthesia/surgery and to being interested in receiving more information about how patients are doing after surgery. Most respondents believed that digital solutions could have made more patients adequately prepared before anaesthesia/surgery and that the number of non‐optimized patients during anaesthesia and surgery could be reduced. In addition, most respondents agreed/strongly agreed that digital solutions could promote the possibilities of adapting the perioperative process to the patients' individual needs and situations. Likewise, most agreed/strongly agreed that patients using digital solutions can lead to reduced costs for the healthcare provider and reduced cancelled anaesthesia/surgeries and that there is an interest in introducing digital solutions for patients to use before anaesthesia/surgery in the workplace.

**TABLE 2:** Responses to the scaled‐response questions, ranging from strongly disagree (1) to strongly agree (5), for all respondents (*n* = 627).

	Strongly disagree	Disagree	Neutral	Agree	Strongly agree	Do not know
Question	*n* (%)	*n* (%)	*n* (%)	*n* (%)	*n* (%)	*n* (%)
Attitudes towards digitalization in the workplace						
I believe that the digital solutions we use in my workplace today have made my work easier.	23 (3.7)	68 (10.8)	125 (19.9)	281 (44.8)	77 (12.3)	53 (8.5)
2 Digital solutions that facilitate the preoperative preparations for a patient could be helpful before anaesthesia/surgery (e.g., virtual reality).	9 (1.4)	21 (3.3)	58 (9.3)	274 (43.7)	99 (15.8)	166 (26.5)
3 Digital solutions that support the patient's preparations for anaesthesia/surgery (e.g., reminders to fill out the health declaration or take premedication) could facilitate my work.	8 (1.3)	14 (2.2)	68 (10.8)	329 (52.5)	139 (22.2)	69 (11.0)
4 Digital solutions can contribute to increased patient involvement during the perioperative process (e.g., with a chat function).	16 (2.6)	31 (4.9)	73 (11.6)	300 (47.8)	97 (15.5)	110 (17.5)
5 Digital solutions are safe to use for patients scheduled for anaesthesia/surgery.	4 (0.6)	31 (4.9)	111 (17.7)	234 (37.3)	67 (10.7)	180 (28.7)
Perceptions of information provision in the workplace						
6 I feel that I am involved in the patients' journey through the perioperative care process.	60 (9.6)	117 (18.7)	108 (17.2)	242 (38.6)	92 (14.7)	8 (1.3)
7 I believe that patients are well‐informed before the preoperative meeting with the anaesthetist.	24 (3.8)	107 (17.1)	136 (21.7)	111 (17.7)	11 (1.8)	238 (38.0)
8 I feel that the patients I meet have received enough information before anaesthesia/surgery.	27 (4.3)	135 (21.5)	169 (27.0)	255 (40.7)	14 (2.2)	27 (4.3)
9 I believe that I receive information about the patients' recovery after anaesthesia/surgery.	276 (44.0)	208 (33.2)	76 (12.1)	49 (7.8)	7 (1.1)	11 (1.8)
10 I believe that I receive information about what happens with the patient in the hospital from the time of anaesthesia until discharge.	354 (56.6)	165 (26.3)	55 (8.8)	33 (5.3)	8 (1.3)	12 (1.9)
11 I believe that I receive information about the patients' rehabilitation after surgery.	443 (70.7)	132 (21.1)	28 (4.5)	11 (1.8)	2 (0.3)	11 (1.8)
12 I feel that I receive information about patients' potential side effects of the anaesthesia and/or surgery.	236 (37.6)	202 (32.2)	96 (15.3)	74 (11.8)	9 (1.4)	10 (1.6)
13 I feel that the patients receive good support in the preparations required for anaesthesia and/or surgery (e.g., fasting, showering, discontinuing medication).	25 (4.0)	92 (14.7)	136 (21.7)	263 (41.9)	21 (3.3)	90 (14.4)
14 My impression is that patients today can easily get in touch with healthcare if they have questions about the anaesthesia/surgery or preoperative preparations.	72 (11.5)	143 (22.8)	94 (15.0)	125 (19.9)	17 (2.7)	176 (28.1)
15 I believe that patients today receive good support in the goals set after anaesthesia/surgery (e.g., exercise and diet).	42 (6.7)	70 (11.2)	88 (14.0)	86 (13.7)	0 (0)	341 (54.4)
16 I am interested in systematically receiving more information about how patients are doing after surgery and have been discharged from the hospital/healthcare provider during the first month.	27 (4.3)	42 (6.7)	98 (15.6)	285 (45.5)	156 (24.9)	19 (3.0)
17 I think it would be beneficial if the patient had an easy way to contact the healthcare provider before and after anaesthesia/surgery (e.g., by sending pictures of gape ability and the surgical wound).	17 (2.7)	42 (6.7)	91 (14.5)	281 (44.8)	141 (22.5)	55 (8.8)
Perceptions of future digitalization in anaesthesia and surgical healthcare						
18 I believe that digital solutions help more patients be adequately prepared for their anaesthesia/surgery.	12 (1.9)	19 (3.0)	73 (11.6)	333 (53.1)	122 (19.5)	68 (10.8)
19 I believe it is possible to reduce the number of non‐optimized patients for anaesthesia/surgery by providing the patients with digital support and reminders about their preoperative preparations and evaluations.	10 (1.6)	21 (3.3)	54 (8.6)	348 (55.5)	150 (23.9)	44 (7.0)
20 Digital solutions could enhance the ability to tailor the perioperative process to the patient's needs and individual situation.	15 (2.4)	19 (3.0)	66 (10.5)	327 (52.2)	107 (17.1)	93 (14.8)
21 I believe that patients who use digital solutions can help create a better flow in the anaesthesia/surgery department (with reduced start and turnover times).	40 (6.4)	60 (9.6)	130 (20.7)	223 (35.6)	89 (14.2)	85 (13.6)
22 I believe that patients who use digital solutions can lead to reduced costs for the healthcare provider.	20 (3.2)	25 (4.0)	100 (15.9)	245 (39.1)	86 (13.7)	151 (24.1)
23 I believe that patients who use digital solutions can lead to a reduction in cancelled anaesthesia/surgeries.	27 (4.3)	33 (5.3)	96 (15.3)	259 (41.3)	100 (15.9)	112 (17.9)
24 I believe there is an interest in introducing digital solutions that patients can use before their anaesthesia/surgery at my workplace.	17 (2.7)	39 (6.2)	86 (13.7)	255 (40.7)	87 (13.9)	143 (22.8)

Independent‐samples Kruskal–Wallis tests were performed to examine the differences in responses to the scaled‐response questions based on respondents' age and the part of Sweden where they worked. Responses of “do not know” were not included in the analyses. There were no statistically significant differences between age and responses regarding attitudes towards digitalization in the workplace (Table S1). However, statistically significant differences to responses to two questions were observed among respondents working in different parts of Sweden (Table 4, Table S2). In the post‐hoc analysis, the largest differences were observed between respondents working in Southern Sweden and the middle of Sweden, where respondents in the middle of Sweden revealed greater confidence in digital solutions contributing to increased patient participation (*p*_adj_ <.001) and that they are safe to use for the patients (*p*_adj_ = .007) compared with respondents working in Southern Sweden.

Regarding perceptions of information provision in the workplace, responses to three questions were statistically significant different between age groups (Table 3, Table S1). The largest differences were between the youngest (20–30 years) and oldest (61–70 years) respondents, with the oldest more strongly believing they receive follow‐up information on patients after anaesthesia (*p*_adj_ = .035) and that patients are well‐supported in preanaesthesia preparations (*p*_adj_ = .011). Additionally, significant differences were seen in relation to what part of Sweden the respondents worked in (Table 4, Table S2). Respondents in Southern Sweden felt more involved in the patient's perioperative journey compared with respondents working in the middle of Sweden (*p*_adj_ = .006). However, respondents in the middle of Sweden showed greater interest in receiving systematic patient updates (*p*_adj_ = .036) and felt more strongly that patients should have easy contact with care providers (*p*_adj_ = .001) compared with respondents working in Southern Sweden.

**TABLE 3:** Statistically significant differences in responses to the scaled‐response questions by respondents' age.

Question	Age	*N*	Mean	SD	Sample average rank	Test statistic^a^	*df*	*p*‐value
Perceptions of information provision in the workplace								
I believe that patients are well‐informed before the preoperative meeting with the anesthetist.	20–30	17	2.47	0.800	138.71	10.334	4	.035
	31–40	102	2.92	0.909	192.73			
	41–50	105	2.84	0.900	184.51			
	51–60	115	3.13	1.047	216.41			
	61–70	50	2.94	0.956	191.55			
	Total	389	2.94	0.959				
	Total	616	1.87	0.988				
I believe that I receive information about what happens with the patient in the hospital from the time of anesthesia until discharge.	20–30	28	1.32	0.612	250.23	11.875	4	.018
	31–40	167	1.68	0.970	310.19			
	41–50	166	1.65	0.873	312.49			
	51–60	176	1.59	0.934	291.44			
	61–70	78	1.92	1.066	351.85			
	Total	615	1.66	0.940				
	Total	617	2.06	1.071				
I feel that the patients receive good support in the preparations required for anesthesia and/or surgery (e.g., fasting, showering, discontinuing medication).	20–30	28	2.79	1.134	193.45	13.567	4	.009
	31–40	147	3.25	0.905	260.68			
	41–50	144	3.26	0.944	260.81			
	51–60	149	3.40	0.986	285.61			
	61–70	69	3.51	0.868	298.60			
	Total	537	3.30	0.956				
	Total	572	3.85	0.973				
Perceptions of future digitalization in anesthesia and surgical healthcare								
I believe that digital solutions help more patients be adequately prepared for their anesthesia/surgery.	20–30	26	4.27	0.533	333.19	9.650	4	.047
	31–40	160	3.98	0.839	285.86			
	41–50	149	4.04	0.706	289.39			
	51–60	152	3.90	0.890	272.74			
	61–70	72	3.74	0.904	243.65			
	Total	559	3.96	0.823				
I believe it is possible to reduce the number of non‐optimized patients for anesthesia/surgery by providing the patients with digital support and reminders about their preoperative preparations and evaluations.	20–30	27	4.48	0.509	379.39	18.829	4	<.001
	31–40	162	3.98	0.870	283.50			
	41–50	151	4.17	0.668	310.16			
	51–60	166	4.03	0.820	289.51			
	61–70	77	3.81	0.874	249.01			
	Total	583	4.04	0.805				
I believe that patients who use digital solutions can help create a better flow in the anesthesia/surgery department (with reduced start and turnover times).	20–30	24	4.13	0.741	357.23	16.317	4	.003
	31–40	155	3.60	1.108	289.08			
	41–50	141	3.45	1.052	261.78			
	51–60	148	3.45	1.162	269.07			
	61–70	74	3.16	1.159	230.26			
	Total	542	3.48	1.116				
I believe that patients who use digital solutions can lead to reduced costs for the healthcare provider.	20–30	23	3.96	0.878	263.15	10.709	4	.030
	31–40	127	3.83	0.918	250.07			
	41–50	126	3.83	0.913	249.06			
	51–60	138	3.71	0.953	234.22			
	61–70	62	3.37	1.075	193.72			
	Total	476	3.74	0.956				
I believe that patients who use digital solutions can lead to a reduction in canceled anesthesia/surgeries.	20–30	24	4.08	0.929	312.31	11.657	4	.020
	31–40	147	3.80	1.033	270.34			
	41–50	135	3.79	0.949	262.96			
	51–60	143	3.66	1.041	251.05			
	61–70	66	3.42	1.039	215.67			
	Total	515	3.72	1.017				

^a^ The test statistic is adjusted for ties.

**TABLE 4:** Statistically significant differences in responses to the scaled‐response questions based on which part of Sweden where respondents worked.

Question	Part of Sweden	*N*	Mean	SD	Sample average rank	Test statistic^a^	df	*p*‐value
Attitudes towards digitalization in the workplace								
Digital solutions can contribute to increased patient involvement during the perioperative process (e.g., with a chat function).	South	249	3.65	0.930	230.04	23.830	2	<.001
	Middle	189	4.05	0.774	291.47			
	North	79	3.89	1.013	272.61			
	Total	517	3.83	0.906				
Digital solutions are safe to use for patients scheduled for anesthesia/surgery.	South	224	3.63	0.793	207.51	9.366	2	.009
	Middle	160	3.88	0.807	244.52			
	North	63	3.76	0.962	230.52			
	Total	447	3.74	0.829				
Perceptions of information provision in the workplace								
I feel that I am involved in the patients' journey through the perioperative care process.	South	304	3.46	1.168	331.42	9.764	2	.008
	Middle	225	3.13	1.234	285.04			
	North	90	3.23	1.255	300.07			
	Total	619	3.31	1.213				
	Total	286	2.76	1.039				
I am interested in systematically receiving more information about how patients are doing after surgery and have been discharged from the hospital/healthcare provider during the first month.	South	300	3.74	1.038	289.57	6.357	2	.042
	Middle	220	3.96	0.981	326.23			
	North	88	3.78	1.108	301.07			
	Total	608	3.82	1.032				
I think it would be beneficial if the patient had an easy way to contact the healthcare provider before and after anesthesia/surgery (e.g., by sending pictures of gape ability and the surgical wound).	South	275	3.72	0.981	262.87	13.659	2	.001
	Middle	211	4.01	0.951	314.33			
	North	86	3.90	0.946	293.78			
	Total	572	3.85	0.973				
Perceptions of future digitalization in anesthesia and surgical healthcare								
I believe that digital solutions help more patients be adequately prepared for their anesthesia/surgery.	South	271	3.88	0.808	264.59	7.399	2	.025
	Middle	202	4.06	0.814	300.56			
	North	86	3.94	0.873	280.28			
	Total	559	3.96	0.823				
Digital solutions could enhance the ability to tailor the perioperative process to the patient's needs and individual situation.	South	255	3.85	0.809	251.76	8.392	2	.015
	Middle	196	4.04	0.809	288.74			
	North	83	3.86	1.002	265.69			
	Total	534	3.92	0.845				
I believe that patients who use digital solutions can lead to reduced costs for the healthcare provider.	South	235	3.63	0.921	221.46	8.410	2	.015
	Middle	169	3.87	0.897	254.64			
	North	72	3.78	1.153	256.25			
	Total	476	3.74	0.956				
I believe that patients who use digital solutions can lead to a reduction in canceled anesthesia/surgeries.	South	250	3.58	1.074	239.61	8.646	2	.013
	Middle	185	3.86	0.926	275.20			
	North	80	3.85	0.982	275.70			
	Total	515	3.72	1.017				

^a^ The test statistic is adjusted for ties.

Out of the seven questions regarding perceptions of future digitalization within anaesthesia and surgical healthcare, five responses were statistically significant different between age groups (Table 3, Table S1) and four between the parts of Sweden (Table 4, Table S2). Post‐hoc analysis on age groups revealed that the biggest differences were observed between the youngest (20–30 years) and the oldest (61–70 years) respondents. Respondents in the youngest age group were more confident that digital support could lead to better preparation of patients prior to anaesthesia/surgery (*p*_adj_ = .001), as well as create a better flow within anaesthesia/surgery (*p*_adj_ = .003), compared with the oldest respondents. Additionally, the youngest respondents believed to a higher degree that patients using digital solutions can reduce cancelled anaesthesia/surgeries compared with the oldest (*p*_adj_ = .033). However, when evaluating whether digital solutions can lead to reduced costs for the healthcare provider, the biggest difference was seen between participants in the age groups 31–40 and 61–70 years, where the younger respondents had a higher belief compared with the oldest (*p*_adj_ = .041). In relation to what part of Sweden the respondents worked, the biggest differences were observed between respondents working in Southern Sweden and the middle of Sweden. Respondents from the middle of Sweden had stronger beliefs that digital solutions can better prepare patients for anaesthesia/surgery (*p*_adj_ = .020), personalize the perioperative process (*p*_adj_ = .011), reduce provider costs (*p*_adj_ = .028), and decrease cancelled anaesthesia/surgeries (*p*_adj_ = .023) compared with respondents in Southern Sweden.

## 4 DISCUSSION

In this study, the perceptions and attitudes of anaesthesia care professionals towards the implementation and advancement of digital solutions in perioperative care were evaluated. Results indicate that anaesthesia personnel are confident that the use of digital solutions may increase the efficiency of care within the anaesthesia setting. Most respondents expressed agreement to digital solutions enhancing their own work efficiency, as well as the preoperative preparations and patient engagement, which is consistent with the results from a previous study.^3^ Regarding the belief that digital solutions are safe to use for patients scheduled for anaesthesia/surgery, there was a statistically significant discrepancy between respondents working in Southern Sweden and the middle of Sweden, where respondents in the middle of Sweden had a higher belief. More than half of the respondents from the middle of Sweden worked in university hospitals, where the personnel may be further educated to a higher degree. However, this cannot be the only reason. Respondents in Northern Sweden also had a higher belief of safety compared with respondents in Southern Sweden, and less than 10% of respondents in Northern Sweden worked in university hospitals. Conversely, there was no statistically significant difference between age groups on the belief of safety. This was somewhat surprising since previous studies have reported that older adults may have a lack of trust in communicative eHealth services.^23^ Additionally, even though approximately half of the respondents agreed/strongly agreed to digital solutions being safe for patients to use, almost one‐third were uncertain of their beliefs. Perhaps knowledge gaps or fear of using digital technology may be reasons to why the personnel could not answer this question.^19^

Most respondents agreed/strongly agreed to being involved in the patient's journey throughout the perioperative care process. However, there was a significant difference between respondents working in Southern Sweden and the middle of Sweden, where respondents in Southern Sweden to a higher degree felt involved in the patient's journey. More respondents in Southern Sweden worked in regional hospitals, compared with respondents in the middle of Sweden, who to a larger extent worked in university hospitals. Generally, university hospitals are larger and work in larger teams than regional hospitals. Hence, personnel in regional hospitals may follow the patients for longer periods than personnel in university hospitals.

Almost 40% of the respondents did not know whether patients were well‐informed before the preoperative meeting with the anaesthetist. This result could perhaps have been surprising, considering that only 2.1% of the respondents worked in a postoperative setting. However, because of the current fast‐paced work environments, personnel may not have enough time to ask, nor listen to, the patients or communicate with other team members.^3^ Additionally, since only approximately 2% worked in a postoperative setting, it was not very surprising that most respondents did not experience receiving information about the patient's recovery, rehabilitation, or possible side effects after anaesthesia/surgery. Even though respondents in all age groups had relatively low agreement, respondents in the 20–30 years age group had the lowest mean scores and respondents in the 61–70 years age group had the highest mean scores. In Sweden, the healthcare system is not structured in such a way that information is easily accessible. Most respondents were, however, interested in systematically receiving more information about how things are going for the patient, that is, information that may be beneficial to enhance preoperative and surgical care. It is also encouraging that the youngest respondents were significantly more interested in getting more information compared with the oldest.

Most respondents believed that digital solutions could improve the patient's readiness for anaesthesia and surgery, ultimately reducing the number of patients who are not adequately prepared for these procedures. These insights align with existing research in public health, indicating that digitalization can empower patients and promote greater participation.^7^ Most respondents also believed that patients using digital solutions can lead to reduced cancelled anaesthesia/surgeries and reduced costs for the care provider, which has also been reported previously.^24^ Respondents in the 20–30 years age group were more confident that digital support could reduce the number of non‐optimized patients, create a better flow within anaesthesia/surgery, promote adaptation of the perioperative process to the patient's needs, reduce costs for the care provider, and reduce cancelled anaesthesia/surgeries compared with the respondents in the 61–70 years age group. Previous studies have reported that age negatively influences the willingness for technical usage.^10^, ^19^ Younger healthcare professionals may be more digitally competent, as well as more comfortable and optimistic regarding new technologies, while older healthcare professionals might have more established routines and be more accustomed to traditional methods, which can make them more cautious or critical of digitalization efforts.^24^ Additionally, personal innovativeness might decrease with age, older people are more likely to be distressed by insufficient technical skills, and older professionals that do not want to learn how to use eHealth systems leave it to the younger.^10^ Subsequently, professionals who are enthusiastic about eHealth may gradually take on more eHealth‐related tasks, which could potentially divide professionals' work roles even further and widen the existing skills gaps in the context of eHealth.^25^ Hence, the motivation and willingness to use digital solutions in patient care are important components of healthcare professionals' digital competence. As digital solutions play a significant role in the implementation process, it is crucial that employees maintain positive attitudes towards digital technologies.^19^, ^26^ Additionally, differences in the perceptions of future digitalization within anaesthesia and surgical healthcare were observed among respondents working in various parts of Sweden, where respondents from the middle of Sweden had higher beliefs in digital solutions compared with respondents in Southern Sweden. Different healthcare settings may have varying levels of resources and infrastructure, and the digital transformation strategy and culture within an institution can vary.^9^ For instance, university hospitals may have better access to digital tools and support systems compared with regional hospitals. Since this variation, based on the healthcare professionals' work settings, may shape their attitudes and responses, organizational support for digitalization is a crucial factor.^25^ Collegial and organizational support are essential factors for building positive experiences of digitalization for healthcare professionals.^19^ Since digitalization can be a useful tool to improve information and education, increase patient participation, and reduce experiences of anxiety and pain in the perioperative context,^27^ it was encouraging that most respondents were interested in introducing digital solutions for patients to utilize prior to their anaesthesia/surgery.

One strength of the current study is that the responses are based on voluntary survey responses from anaesthesiologists and nurse anaesthetists, with an overall response rate of 64.0%. However, it is not possible to state whether the results fully represent the views of the anaesthesia community practice in Sweden. Even though the survey was available through website and email addresses, we cannot discriminate the number of active colleagues who participate in clinical practice. Still, more than 600 responses were compiled and analyzed and should therefore provide a reasonable up‐to‐date profile. One limitation, however, is that no preexisting validated instrument was identified for adaptation to the study. Hence, the development of a new survey was deemed more appropriate. The design of the survey was inspired by the validated KAP survey model,^22^ which tends to favor a more positive construction of statements. Consequently, most items in the survey (though not all) were presented positively, potentially introducing response bias and affecting the quality and accuracy of the collected data. To mitigate this issue, a pilot study was conducted with a small sample of respondents representative of the larger population^28^—seven nurses and five anaesthesiologists—on two occasions, and the wording was adjusted accordingly. The subsequent survey achieved a relatively high response rate (*n* = 627), supporting the validity of the results. However, the subsequent responses may also potentially be based on different understandings of digitalization and digital solutions,^19^ which could obscure what the respondents' responses truly reflect. To clarify the concepts of digitization, digitalization and digital aids/solutions, and minimize discrepancies, an introductory text was provided to the respondents before they took the survey. Another limitation may be the lack of randomization in the order of the questions. Previous findings have highlighted the importance of interpreting associations between different indicators in the context of where they are presented in a survey.^29^ On the contrary, respondents in the current study had the option of answering the questions in the online survey in any preferred order, allowing them to revisit and adjust their responses. If the survey's purpose is exploratory or aims to understand attitudes and opinions in‐depth, allowing participants to modify their responses might add value. However, in standardized or time‐sensitive surveys where consistency is paramount, limiting changes might yield cleaner data.^30^

The findings of this research study have several implications for clinical practice. For successful implementation of digital solutions in anaesthesia and perioperative care, it is essential to consider varying infrastructure and resource availability across the healthcare settings. Digitalization efforts should be adapted to meet the needs of diverse healthcare environments, including urban and rural hospitals, as well as specialized departments. A supportive organizational culture and investment in digital training are also critical to foster positive attitudes and enhance digital competence among healthcare personnel. By providing user‐friendly and efficient digital tools, healthcare institutions could potentially optimize perioperative care, empower patient participation, and ultimately contribute to cost reductions and fewer surgical cancellations.

## 5 CONCLUSION

Swedish anaesthesia personnel are confident that the use of digital solutions can strengthen the efficiency of care within the anaesthesiologic setting and that digital solutions can enhance the exchange of information between patients and healthcare staff, potentially leading to health economic benefits and ensuring evidence‐based care. There are indications that anaesthesia personnel have varying perceptions regarding the benefits and necessity of digital solutions depending on their age and what part of Sweden they work in. These variations of how healthcare professionals perceive the impact, usefulness, and challenges of digital solutions in their field could reinforce tailored digitalization strategies in healthcare. However, further research on various digital solutions and the underlying reasons for differing perceptions of these solutions is needed.

## AUTHOR CONTRIBUTIONS

*Conceptualization and supervision*: PJ and AA. *Methodology*: PJ, CV, KHE, KHA, RS, and AA. *Software and visualization*: AA. *Validation*: PJ, CS, KHE, KHA, RS, and AA. *Formal analysis and writing—original draft preparation*: PJ, CV, and AA. *Writing—review and editing*: PJ, CV, CS, RS, and AA. All authors have read and agreed to the published version of the manuscript.

## Supporting information


Table S1.

Table S2.

## Data Availability

The data that support the findings of this study are available from the corresponding author upon reasonable request.
