# Supplementary material for: Perceptions of eHealth and digitalization among professional anaesthesia personnel: A Swedish national study
Source: Acta Anaesthesiol Scand. 2025 Jan 30;69(3):e14587. doi: 10.1111/aas.14587 (PMC11781013; doi:10.1111/aas.14587)
Supplement: Supplementary file 1 — Table S1. [file AAS-69-0-s002.docx]

**Supplementary Table 1** Responses to the scaled-response questions by respondents’ age

| **Question** | **Age** | ***N*** | **Mean** | **SD** | **Sample average rank** | **Test statistic^a^** | **df** | ***P*-value** |
| --- | --- | --- | --- | --- | --- | --- | --- | --- |
| **Attitudes towards digitalization in the workplace** | | | | | | | | |
| 1. I believe that the digital solutions we use in my workplace today have made my work easier. | 20-30 | 25 | 3.52 | 0.963 | 280.98 | 0.452 | 4 | 0.978 |
|  | 31-40 | 151 | 3.58 | 0.920 | 286.90 |  |  |  |
|  | 41-50 | 147 | 3.52 | 0.995 | 282.10 |  |  |  |
|  | 51-60 | 173 | 3.56 | 1.075 | 290.32 |  |  |  |
|  | 61-70 | 78 | 3.60 | 0.998 | 294.67 |  |  |  |
|  | Total | 574 | 3.56 | 0.997 |  |  |  |  |
| 1. Digital solutions that facilitate the preoperative preparations for a patient could be helpful before anaesthesia/surgery (e.g. Virtual Reality). | 20-30 | 24 | 4.00 | 0.885 | 246.60 | 7.864 | 4 | 0.097 |
|  | 31-40 | 125 | 3.88 | 0.809 | 219.67 |  |  |  |
|  | 41-50 | 118 | 4.02 | 0.877 | 245.39 |  |  |  |
|  | 51-60 | 133 | 4.01 | 0.783 | 239.53 |  |  |  |
|  | 61-70 | 61 | 3.74 | 0.874 | 201.64 |  |  |  |
|  | Total | 461 | 3.94 | 0.834 |  |  |  |  |
| 1. Digital solutions that support the patient’s preparations for anaesthesia/surgery (e.g. reminders to fill out the health declaration or take premedication) could facilitate my work. | 20-30 | 27 | 4.22 | 0.641 | 315.46 | 8.118 | 4 | 0.087 |
|  | 31-40 | 154 | 3.99 | 0.820 | 275.62 |  |  |  |
|  | 41-50 | 147 | 4.15 | 0.715 | 300.10 |  |  |  |
|  | 51-60 | 158 | 4.01 | 0.723 | 270.00 |  |  |  |
|  | 61-70 | 72 | 3.86 | 0.909 | 253.08 |  |  |  |
|  | Total | 558 | 4.03 | 0.775 |  |  |  |  |
| 1. Digital solutions can contribute to increased patient involvement during the perioperative process (e.g. with a chat function). | 20-30 | 24 | 3.71 | 1.042 | 242.21 | 6.860 | 4 | 0.143 |
|  | 31-40 | 139 | 3.91 | 0.921 | 275.31 |  |  |  |
|  | 41-50 | 137 | 3.84 | 0.842 | 256.85 |  |  |  |
|  | 51-60 | 146 | 3.88 | 0.859 | 263.92 |  |  |  |
|  | 61-70 | 71 | 3.61 | 1.021 | 226.77 |  |  |  |
|  | Total | 517 | 3.83 | 0.906 |  |  |  |  |
| 1. Digital solutions are safe to use for patients scheduled for anaesthesia/surgery. | 20-30 | 23 | 4.00 | 0.603 | 259.67 | 4.680 | 4 | 0.322 |
|  | 31-40 | 124 | 3.65 | 0.857 | 212.95 |  |  |  |
|  | 41-50 | 119 | 3.82 | 0.788 | 235.78 |  |  |  |
|  | 51-60 | 123 | 3.72 | 0.792 | 218.20 |  |  |  |
|  | 61-70 | 58 | 3.67 | 0.980 | 221.62 |  |  |  |
|  | Total | 447 | 3.74 | 0.829 |  |  |  |  |
| **Perceptions of information provision in the workplace** | | | | | | | | |
| 1. I feel that I am involved in the patients’ journey through the perioperative care process. | 20-30 | 28 | 3.21 | 1.228 | 294.98 | 3.934 | 4 | 0.415 |
|  | 31-40 | 169 | 3.32 | 1.212 | 312.78 |  |  |  |
|  | 41-50 | 168 | 3.23 | 1.246 | 299.29 |  |  |  |
|  | 51-60 | 176 | 3.27 | 1.230 | 305.28 |  |  |  |
|  | 61-70 | 78 | 3.55 | 1.089 | 343.10 |  |  |  |
|  | Total | 619 | 3.31 | 1.213 |  |  |  |  |
| 1. I believe that patients are well-informed before the preoperative meeting with the anaesthetist. | 20-30 | 17 | 2.47 | 0.800 | 138.71 | 10.334 | 4 | 0.035 |
|  | 31-40 | 102 | 2.92 | 0.909 | 192.73 |  |  |  |
|  | 41-50 | 105 | 2.84 | 0.900 | 184.51 |  |  |  |
|  | 51-60 | 115 | 3.13 | 1.047 | 216.41 |  |  |  |
|  | 61-70 | 50 | 2.94 | 0.956 | 191.55 |  |  |  |
|  | Total | 389 | 2.94 | 0.959 |  |  |  |  |
| 1. I feel that the patients I meet have received enough information before anaesthesia/surgery. | 20-30 | 28 | 2.75 | 1.041 | 235.89 | 7.299 | 4 | 0.121 |
|  | 31-40 | 166 | 3.14 | 0.927 | 296.40 |  |  |  |
|  | 41-50 | 164 | 3.13 | 0.852 | 291.87 |  |  |  |
|  | 51-60 | 167 | 3.23 | 1.035 | 317.23 |  |  |  |
|  | 61-70 | 75 | 3.24 | 0.942 | 315.31 |  |  |  |
|  | Total | 600 | 3.16 | 0.949 |  |  |  |  |
| 1. I believe that I receive information about the patients’ recovery after anaesthesia/surgery. | 20-30 | 28 | 1.75 | 0.928 | 288.59 | 8.169 | 4 | 0.086 |
|  | 31-40 | 168 | 1.86 | 0.902 | 314.70 |  |  |  |
|  | 41-50 | 165 | 1.88 | 0.990 | 311.36 |  |  |  |
|  | 51-60 | 176 | 1.76 | 0.997 | 285.88 |  |  |  |
|  | 61-70 | 79 | 2.13 | 1.125 | 346.80 |  |  |  |
|  | Total | 616 | 1.87 | 0.988 |  |  |  |  |
| 1. I believe that I receive information about what happens with the patient in the hospital from the time of anaesthesia until discharge. | 20-30 | 28 | 1.32 | 0.612 | 250.23 | 11.875 | 4 | 0.018 |
|  | 31-40 | 167 | 1.68 | 0.970 | 310.19 |  |  |  |
|  | 41-50 | 166 | 1.65 | 0.873 | 312.49 |  |  |  |
|  | 51-60 | 176 | 1.59 | 0.934 | 291.44 |  |  |  |
|  | 61-70 | 78 | 1.92 | 1.066 | 351.85 |  |  |  |
|  | Total | 615 | 1.66 | 0.940 |  |  |  |  |
| 1. I believe that I receive information about the patients’ rehabilitation after surgery. | 20-30 | 28 | 1.14 | 0.356 | 263.07 | 8.599 | 4 | 0.072 |
|  | 31-40 | 168 | 1.35 | 0.639 | 306.19 |  |  |  |
|  | 41-50 | 165 | 1.38 | 0.676 | 311.81 |  |  |  |
|  | 51-60 | 176 | 1.32 | 0.624 | 299.40 |  |  |  |
|  | 61-70 | 79 | 1.59 | 0.941 | 342.87 |  |  |  |
|  | Total | 616 | 1.37 | 0.687 |  |  |  |  |
| 1. I feel that I receive information about patients’ potential side effects of the anaesthesia and/or surgery. | 20-30 | 28 | 1.79 | 0.787 | 275.79 | 6.637 | 4 | 0.156 |
|  | 31-40 | 169 | 2.02 | 0.932 | 312.12 |  |  |  |
|  | 41-50 | 167 | 2.11 | 1.092 | 316.86 |  |  |  |
|  | 51-60 | 176 | 1.98 | 1.141 | 289.60 |  |  |  |
|  | 61-70 | 77 | 2.30 | 1.204 | 341.53 |  |  |  |
|  | Total | 617 | 2.06 | 1.071 |  |  |  |  |
| 1. I feel that the patients receive good support in the preparations required for anaesthesia and/or surgery (e.g. fasting, showering, discontinuing medication). | 20-30 | 28 | 2.79 | 1.134 | 193.45 | 13.567 | 4 | 0.009 |
|  | 31-40 | 147 | 3.25 | 0.905 | 260.68 |  |  |  |
|  | 41-50 | 144 | 3.26 | 0.944 | 260.81 |  |  |  |
|  | 51-60 | 149 | 3.40 | 0.986 | 285.61 |  |  |  |
|  | 61-70 | 69 | 3.51 | 0.868 | 298.60 |  |  |  |
|  | Total | 537 | 3.30 | 0.956 |  |  |  |  |
| 1. My impression is that patients today can easily get in touch with healthcare if they have questions about the anaesthesia/surgery or preoperative preparations. | 20-30 | 22 | 2.18 | 1.006 | 167.32 | 7.791 | 4 | 0.100 |
|  | 31-40 | 122 | 2.83 | 1.111 | 237.51 |  |  |  |
|  | 41-50 | 117 | 2.63 | 1.134 | 217.26 |  |  |  |
|  | 51-60 | 130 | 2.70 | 1.192 | 224.11 |  |  |  |
|  | 61-70 | 60 | 2.88 | 1.121 | 245.26 |  |  |  |
|  | Total | 451 | 2.72 | 1.143 |  |  |  |  |
| 1. I believe that patients today receive good support in the goals set after anaesthesia/surgery (e.g. exercise and diet). | 20-30 | 17 | 2.29 | 1.047 | 108.44 | 3.922 | 4 | 0.417 |
|  | 31-40 | 83 | 2.82 | 1.026 | 147.60 |  |  |  |
|  | 41-50 | 72 | 2.82 | 1.039 | 147.99 |  |  |  |
|  | 51-60 | 75 | 2.72 | 1.073 | 140.71 |  |  |  |
|  | 61-70 | 39 | 2.82 | 0.997 | 147.14 |  |  |  |
|  | Total | 286 | 2.76 | 1.039 |  |  |  |  |
| 1. I am interested in systematically receiving more information about how patients are doing after surgery and have been discharged from the hospital/healthcare provider during the first month. | 20-30 | 28 | 4.18 | 0.819 | 359.93 | 8.609 | 4 | 0.072 |
|  | 31-40 | 166 | 3.92 | 0.969 | 318.11 |  |  |  |
|  | 41-50 | 163 | 3.91 | 0.942 | 312.61 |  |  |  |
|  | 51-60 | 172 | 3.68 | 1.112 | 284.32 |  |  |  |
|  | 61-70 | 79 | 3.65 | 1.166 | 283.44 |  |  |  |
|  | Total | 608 | 3.82 | 1.032 |  |  |  |  |
| 1. I think it would be beneficial if the patient had an easy way to contact the healthcare provider before and after anaesthesia/surgery (e.g., by sending pictures of gape ability and the surgical wound). | 20-30 | 24 | 3.83 | 1.274 | 297.90 | 4.984 | 4 | 0.289 |
|  | 31-40 | 158 | 3.92 | 0.937 | 297.33 |  |  |  |
|  | 41-50 | 151 | 3.94 | 0.911 | 299.14 |  |  |  |
|  | 51-60 | 164 | 3.77 | 1.025 | 274.15 |  |  |  |
|  | 61-70 | 75 | 3.72 | 0.938 | 261.59 |  |  |  |
|  | Total | 572 | 3.85 | 0.973 |  |  |  |  |
| **Perceptions of future digitalization in anaesthesia and surgical healthcare** | | | | | | | | |
| 1. I believe that digital solutions help more patients be adequately prepared for their anaesthesia/surgery. | 20-30 | 26 | 4.27 | 0.533 | 333.19 | 9.650 | 4 | 0.047 |
|  | 31-40 | 160 | 3.98 | 0.839 | 285.86 |  |  |  |
|  | 41-50 | 149 | 4.04 | 0.706 | 289.39 |  |  |  |
|  | 51-60 | 152 | 3.90 | 0.890 | 272.74 |  |  |  |
|  | 61-70 | 72 | 3.74 | 0.904 | 243.65 |  |  |  |
|  | Total | 559 | 3.96 | 0.823 |  |  |  |  |
| 1. I believe it is possible to reduce the number of non-optimized patients for anaesthesia/surgery by providing the patients with digital support and reminders about their preoperative preparations and evaluations. | 20-30 | 27 | 4.48 | 0.509 | 379.39 | 18.829 | 4 | <0.001 |
|  | 31-40 | 162 | 3.98 | 0.870 | 283.50 |  |  |  |
|  | 41-50 | 151 | 4.17 | 0.668 | 310.16 |  |  |  |
|  | 51-60 | 166 | 4.03 | 0.820 | 289.51 |  |  |  |
|  | 61-70 | 77 | 3.81 | 0.874 | 249.01 |  |  |  |
|  | Total | 583 | 4.04 | 0.805 |  |  |  |  |
| 1. Digital solutions could enhance the ability to tailor the perioperative process to the patient’s needs and individual situation. | 20-30 | 25 | 4.12 | 0.881 | 307.48 | 9.192 | 4 | 0.056 |
|  | 31-40 | 147 | 3.90 | 0.912 | 268.00 |  |  |  |
|  | 41-50 | 141 | 4.02 | 0.638 | 274.44 |  |  |  |
|  | 51-60 | 148 | 3.95 | 0.863 | 273.21 |  |  |  |
|  | 61-70 | 73 | 3.66 | 0.961 | 227.84 |  |  |  |
|  | Total | 534 | 3.92 | 0.845 |  |  |  |  |
| 1. I believe that patients who use digital solutions can help create a better flow in the anaesthesia/surgery department (with reduced start and turnover times). | 20-30 | 24 | 4.13 | 0.741 | 357.23 | 16.317 | 4 | 0.003 |
|  | 31-40 | 155 | 3.60 | 1.108 | 289.08 |  |  |  |
|  | 41-50 | 141 | 3.45 | 1.052 | 261.78 |  |  |  |
|  | 51-60 | 148 | 3.45 | 1.162 | 269.07 |  |  |  |
|  | 61-70 | 74 | 3.16 | 1.159 | 230.26 |  |  |  |
|  | Total | 542 | 3.48 | 1.116 |  |  |  |  |
| 1. I believe that patients who use digital solutions can lead to reduced costs for the healthcare provider. | 20-30 | 23 | 3.96 | 0.878 | 263.15 | 10.709 | 4 | 0.030 |
|  | 31-40 | 127 | 3.83 | 0.918 | 250.07 |  |  |  |
|  | 41-50 | 126 | 3.83 | 0.913 | 249.06 |  |  |  |
|  | 51-60 | 138 | 3.71 | 0.953 | 234.22 |  |  |  |
|  | 61-70 | 62 | 3.37 | 1.075 | 193.72 |  |  |  |
|  | Total | 476 | 3.74 | 0.956 |  |  |  |  |
| 1. I believe that patients who use digital solutions can lead to a reduction in cancelled anaesthesia/surgeries. | 20-30 | 24 | 4.08 | 0.929 | 312.31 | 11.657 | 4 | 0.020 |
|  | 31-40 | 147 | 3.80 | 1.033 | 270.34 |  |  |  |
|  | 41-50 | 135 | 3.79 | 0.949 | 262.96 |  |  |  |
|  | 51-60 | 143 | 3.66 | 1.041 | 251.05 |  |  |  |
|  | 61-70 | 66 | 3.42 | 1.039 | 215.67 |  |  |  |
|  | Total | 515 | 3.72 | 1.017 |  |  |  |  |
| 1. I believe there is an interest in introducing digital solutions that patients can use before their anaesthesia/surgery at my workplace. | 20-30 | 24 | 3.96 | 0.806 | 267.52 | 1.847 | 4 | 0.764 |
|  | 31-40 | 150 | 3.71 | 0.999 | 240.37 |  |  |  |
|  | 41-50 | 124 | 3.67 | 1.010 | 234.27 |  |  |  |
|  | 51-60 | 122 | 3.80 | 0.909 | 249.56 |  |  |  |
|  | 61-70 | 64 | 3.72 | 0.951 | 240.61 |  |  |  |
|  | Total | 484 | 3.74 | 0.963 |  |  |  |  |

^a^The test statistic is adjusted for ties.
