# Supplementary material for: Perceptions of eHealth and digitalization among professional anaesthesia personnel: A Swedish national study
Source: Acta Anaesthesiol Scand. 2025 Jan 30;69(3):e14587. doi: 10.1111/aas.14587 (PMC11781013; doi:10.1111/aas.14587)
Supplement: Supplementary file 2 — Table S2. [file AAS-69-0-s001.docx]

**Supplementary Table 2** Responses to the scaled-response questions based on which part of Sweden where respondents worked

| **Question** | **Part of Sweden** | ***N*** | **Mean** | **SD** | **Sample average rank** | **Test statistic^a^** | **df** | ***P*-value** |
| --- | --- | --- | --- | --- | --- | --- | --- | --- |
| **Attitudes towards digitalization in the workplace** | | | | | | | | |
| 1. I believe that the digital solutions we use at my workplace today have made my work easier. | South | 283 | 3.52 | 0.972 | 280.06 | 1.333 | 2 | 0.513 |
|  | Middle | 204 | 3.60 | 1.029 | 295.91 |  |  |  |
|  | North | 87 | 3.59 | 1.006 | 291.98 |  |  |  |
|  | Total | 574 | 3.56 | 0.997 |  |  |  |  |
| 1. Digital solutions that facilitate the preoperative preparations for a patient could be helpful before anesthesia/surgery (e.g., Virtual Reality). | South | 229 | 3.87 | 0.795 | 218.34 | 5.383 | 2 | 0.068 |
|  | Middle | 160 | 4.01 | 0.890 | 245.18 |  |  |  |
|  | North | 72 | 4.00 | 0.822 | 239.75 |  |  |  |
|  | Total | 461 | 3.94 | 0.834 |  |  |  |  |
| 1. Digital solutions that support the patient’s preparations for anesthesia/surgery (e.g., reminders to fill out the health declaration or take premedication) could facilitate my work. | South | 272 | 4.00 | 0.731 | 270.60 | 2.140 | 2 | 0.343 |
|  | Middle | 202 | 4.10 | 0.719 | 289.29 |  |  |  |
|  | North | 84 | 3.98 | 1.006 | 284.77 |  |  |  |
|  | Total | 558 | 4.03 | 0.775 |  |  |  |  |
| 1. Digital solutions can contribute to increased patient involvement during the perioperative process (e.g., with a chat function). | South | 249 | 3.65 | 0.930 | 230.04 | 23.830 | 2 | <0.001 |
|  | Middle | 189 | 4.05 | 0.774 | 291.47 |  |  |  |
|  | North | 79 | 3.89 | 1.013 | 272.61 |  |  |  |
|  | Total | 517 | 3.83 | 0.906 |  |  |  |  |
| 1. Digital solutions are safe to use for patients scheduled for anesthesia/surgery. | South | 224 | 3.63 | 0.793 | 207.51 | 9.366 | 2 | 0.009 |
|  | Middle | 160 | 3.88 | 0.807 | 244.52 |  |  |  |
|  | North | 63 | 3.76 | 0.962 | 230.52 |  |  |  |
|  | Total | 447 | 3.74 | 0.829 |  |  |  |  |
| **Perceptions of information provision in the workplace** | | | | | | | | |
| 1. I feel that I am involved in the patients’ journey through the perioperative care process. | South | 304 | 3.46 | 1.168 | 331.42 | 9.764 | 2 | 0.008 |
|  | Middle | 225 | 3.13 | 1.234 | 285.04 |  |  |  |
|  | North | 90 | 3.23 | 1.255 | 300.07 |  |  |  |
|  | Total | 619 | 3.31 | 1.213 |  |  |  |  |
| 1. I believe that patients are well-informed before the preoperative meeting with the anesthetist. | South | 183 | 3.03 | 0.870 | 204.34 | 2.652 | 2 | 0.266 |
|  | Middle | 142 | 2.87 | 1.012 | 185.68 |  |  |  |
|  | North | 64 | 2.86 | 1.067 | 188.99 |  |  |  |
|  | Total | 389 | 2.94 | 0.959 |  |  |  |  |
| 1. I feel that the patients I meet have received enough information before anesthesia/surgery. | South | 292 | 3.22 | 0.909 | 310.21 | 3.662 | 2 | 0.160 |
|  | Middle | 217 | 3.14 | 0.968 | 299.06 |  |  |  |
|  | North | 91 | 2.98 | 1.011 | 272.79 |  |  |  |
|  | Total | 600 | 3.16 | 0.949 |  |  |  |  |
| 1. I believe that I receive information about the patients’ recovery after anesthesia/surgery. | South | 301 | 1.81 | 0.957 | 298.55 | 2.115 | 2 | 0.347 |
|  | Middle | 223 | 1.93 | 1.020 | 317.98 |  |  |  |
|  | North | 92 | 1.92 | 1.008 | 318.07 |  |  |  |
|  | Total | 616 | 1.87 | 0.988 |  |  |  |  |
| 1. I believe that I receive information about what happens with the patient in the hospital from the time of anesthesia until discharge. | South | 302 | 1.61 | 0.900 | 299.96 | 2.986 | 2 | 0.225 |
|  | Middle | 223 | 1.76 | 1.015 | 322.57 |  |  |  |
|  | North | 90 | 1.59 | 0.860 | 298.88 |  |  |  |
|  | Total | 615 | 1.66 | 0.940 |  |  |  |  |
| 1. I believe that I receive information about the patients’ rehabilitation after surgery. | South | 303 | 1.40 | 0.697 | 315.33 | 1.611 | 2 | 0.447 |
|  | Middle | 221 | 1.33 | 0.672 | 299.67 |  |  |  |
|  | North | 92 | 1.36 | 0.689 | 307.22 |  |  |  |
|  | Total | 616 | 1.37 | 0.687 |  |  |  |  |
| 1. I feel that I receive information about patients’ potential side effects of the anesthesia and/or surgery. | South | 303 | 2.09 | 1.091 | 314.05 | 0.532 | 2 | 0.767 |
|  | Middle | 222 | 2.01 | 1.025 | 303.79 |  |  |  |
|  | North | 92 | 2.05 | 1.123 | 304.95 |  |  |  |
|  | Total | 617 | 2.06 | 1.071 |  |  |  |  |
| 1. I feel that the patients receive good support in the preparations required for anesthesia and/or surgery (e.g., fasting, showering, discontinuing medication). | South | 262 | 3.35 | 0.946 | 276.25 | 1.305 | 2 | 0.521 |
|  | Middle | 192 | 3.26 | 0.967 | 262.59 |  |  |  |
|  | North | 83 | 3.27 | 0.964 | 260.95 |  |  |  |
|  | Total | 537 | 3.30 | 0.956 |  |  |  |  |
| 1. My impression is that patients today can easily get in touch with healthcare if they have questions about the anesthesia/surgery or preoperative preparations. | South | 215 | 2.82 | 1.102 | 237.69 | 5.037 | 2 | 0.081 |
|  | Middle | 163 | 2.69 | 1.141 | 222.06 |  |  |  |
|  | North | 73 | 2.48 | 1.237 | 200.36 |  |  |  |
|  | Total | 451 | 2.72 | 1.143 |  |  |  |  |
| 1. I believe that patients today receive good support in the goals set after anesthesia/surgery (e.g., exercise and diet). | South | 128 | 2.89 | 0.966 | 152.70 | 4.450 | 2 | 0.108 |
|  | Middle | 105 | 2.72 | 1.105 | 141.29 |  |  |  |
|  | North | 53 | 2.53 | 1.049 | 125.67 |  |  |  |
|  | Total | 286 | 2.76 | 1.039 |  |  |  |  |
| 1. I am interested in systematically receiving more information about how patients are doing after surgery and have been discharged from the hospital/healthcare provider during the first month. | South | 300 | 3.74 | 1.038 | 289.57 | 6.357 | 2 | 0.042 |
|  | Middle | 220 | 3.96 | 0.981 | 326.23 |  |  |  |
|  | North | 88 | 3.78 | 1.108 | 301.07 |  |  |  |
|  | Total | 608 | 3.82 | 1.032 |  |  |  |  |
| 1. I think it would be beneficial if the patient had an easy way to contact the healthcare provider before and after anesthesia/surgery (e.g., by sending pictures of gape ability and the surgical wound). | South | 275 | 3.72 | 0.981 | 262.87 | 13.659 | 2 | 0.001 |
|  | Middle | 211 | 4.01 | 0.951 | 314.33 |  |  |  |
|  | North | 86 | 3.90 | 0.946 | 293.78 |  |  |  |
|  | Total | 572 | 3.85 | 0.973 |  |  |  |  |
| **Perceptions of future digitalization in anesthesia and surgical healthcare** | | | | | | | | |
| 1. I believe that digital solutions help more patients be adequately prepared for their anesthesia/surgery. | South | 271 | 3.88 | 0.808 | 264.59 | 7.399 | 2 | 0.025 |
|  | Middle | 202 | 4.06 | 0.814 | 300.56 |  |  |  |
|  | North | 86 | 3.94 | 0.873 | 280.28 |  |  |  |
|  | Total | 559 | 3.96 | 0.823 |  |  |  |  |
| 1. I believe it is possible to reduce the number of non-optimized patients for anesthesia/surgery by providing the patients with digital support and reminders about their preoperative preparations and evaluations. | South | 282 | 4.00 | 0.800 | 283.37 | 1.864 | 2 | 0.394 |
|  | Middle | 216 | 4.09 | 0.782 | 300.13 |  |  |  |
|  | North | 85 | 4.06 | 0.878 | 299.99 |  |  |  |
|  | Total | 583 | 4.04 | 0.805 |  |  |  |  |
| 1. Digital solutions could enhance the ability to tailor the perioperative process to the patient’s needs and individual situation. | South | 255 | 3.85 | 0.809 | 251.76 | 8.392 | 2 | 0.015 |
|  | Middle | 196 | 4.04 | 0.809 | 288.74 |  |  |  |
|  | North | 83 | 3.86 | 1.002 | 265.69 |  |  |  |
|  | Total | 534 | 3.92 | 0.845 |  |  |  |  |
| 1. I believe that patients who use digital solutions can help create a better flow in the anesthesia/surgery department (with reduced start and turnover times). | South | 265 | 3.40 | 1.079 | 259.28 | 3.752 | 2 | 0.153 |
|  | Middle | 195 | 3.54 | 1.154 | 280.07 |  |  |  |
|  | North | 82 | 3.61 | 1.130 | 290.60 |  |  |  |
|  | Total | 542 | 3.48 | 1.116 |  |  |  |  |
| 1. I believe that patients who use digital solutions can lead to reduced costs for the healthcare provider. | South | 235 | 3.63 | 0.921 | 221.46 | 8.410 | 2 | 0.015 |
|  | Middle | 169 | 3.87 | 0.897 | 254.64 |  |  |  |
|  | North | 72 | 3.78 | 1.153 | 256.25 |  |  |  |
|  | Total | 476 | 3.74 | 0.956 |  |  |  |  |
| 1. I believe that patients who use digital solutions can lead to a reduction in canceled anesthesia/surgeries. | South | 250 | 3.58 | 1.074 | 239.61 | 8.646 | 2 | 0.013 |
|  | Middle | 185 | 3.86 | 0.926 | 275.20 |  |  |  |
|  | North | 80 | 3.85 | 0.982 | 275.70 |  |  |  |
|  | Total | 515 | 3.72 | 1.017 |  |  |  |  |
| 1. I believe there is an interest in introducing digital solutions that patients can use before their anesthesia/surgery at my workplace. | South | 239 | 3.73 | 0.947 | 242.18 | 0.015 | 2 | 0.993 |
|  | Middle | 171 | 3.75 | 0.989 | 243.40 |  |  |  |
|  | North | 74 | 3.73 | 0.969 | 241.46 |  |  |  |
|  | Total | 484 | 3.74 | 0.963 |  |  |  |  |

^a^The test statistic is adjusted for ties.
